# Collagen at the maternal-fetal interface in human pregnancy

**DOI:** 10.7150/ijbs.45586

**Published:** 2020-05-25

**Authors:** Jia-Wei Shi, Zhen-Zhen Lai, Hui-Li Yang, Shao-Liang Yang, Cheng-Jie Wang, Deng Ao, Lu-Yu Ruan, Hui-Hui Shen, Wen-Jie Zhou, Jie Mei, Qiang Fu, Ming-Qing Li

**Affiliations:** 1 NHC Key Lab of Reproduction Regulation (Shanghai Institute of Planned Parenthood Research), Hospital of Obstetrics and Gynecology, Fudan University, Shanghai 200080, People's Republic of China.; 2 Shanghai Key Laboratory of Female Reproductive Endocrine Related Diseases, Hospital of Obstetrics and Gynecology, Fudan University, Shanghai 200080, People's Republic of China.; 3 Center of Reproductive Medicine of Ruijin Hospital, Shanghai Jiao Tong University School of Medicine, Shanghai 200025, People's Republic of China.; 4 Reproductive Medicine Center, Department of Obstetrics and Gynecology, Nanjing Drum Tower Hospital, The Affiliated Hospital of Nanjing University Medical School, Nanjing, Jiangsu 210008, People's Republic of China.; 5 Department of Immunology, Binzhou Medical College, Yantai, 264003, People's Republic of China.

**Keywords:** collagen, NC1 domain, decidua, miscarriage, preeclampsia, diabetes, NK cell, cervix

## Abstract

The survival and development of a semi-allogenic fetus during pregnancy require special immune tolerance microenvironment at the maternal fetal interface. During the establishment of a successful pregnancy, the endometrium undergoes a series of changes, and the extracellular matrix (ECM) breaks down and remodels. Collagen is one of the most abundant ECM. Emerging evidence has shown that collagen and its fragment are expressed at the maternal fetal interface. The regulation of expression of collagen is quite complex, and this process involves a multitude of factors. Collagen exerts a critical role during the successful pregnancy. In addition, the abnormal expressions of collagen and its fragments are associated with certain pathological states associated with pregnancy, including recurrent miscarriage, diabetes mellitus with pregnancy, preeclampsia and so on. In this review, the expression and potential roles of collagen under conditions of physiological and pathological pregnancy are systematically discussed.

## Introduction

For a successful pregnancy, the semi-allogenic fetus must not be rejected by the maternal immune system, and the mechanisms that underlie in this process are of critical importance. The cycling endometrium needs extensive tissue remodeling to prepare the embryo for implantation. Some factors expressed at the maternal fetal interface contribute to the successful implantation and pregnancy.

During the establishment of pregnancy, endometrium undergoes rapid growth and differentiation, extracellular matrix (ECM) breaks down and remodels 1. It is reported that ECM could influence and regulate trophoblast invasion and participate in the remodeling of the decidua at the maternal fetal interface 2,3. Collagen is the one of the most abundant component of ECM, regulating cellular biological behavior and providing the structural integrity of human tissue 4,5. Additionally, collagen is widely distributed in various tissues and organs. Its main function is to construct three-dimensional scaffold to maintain tissue integrity and mediate cell adhesion.

To date, the collagen superfamily comprises 29 types in vertebrates, which are formed by at least 46 different polypeptide chains (α chains) 6. Each collagen is comprised of three α chains with left-handed helix configuration, and the chains are intertwined to form right-handed super helix structure 7. Collagen is mainly degraded by collagenase, which generally exists in tissue in inactive form. Collagen metabolism is related to the expression and activity of matrix metalloproteinase (MMP) 8. The matrix metalloproteinases (MMPs), a family of zinc-dependent proteases, is able to degrade different components of the ECM and plays a crucial role in the remodeling of various tissues and organs. And the MMP family includes collagenases, stromelysins, matrilysins and other MMPs 8. MMP-2 and MMP-9 have an important role in endometrial tissue remodeling during pregnancy. They are thought to be playing a crucial role in pregnancy, because they are able to degrade components of the ECM thereby facilitating cell migration and angiogenesis 8,9.

The maternal fetal interface present different types of collagen and corresponding receptors, including collagen type I, III, IV and so on 10. And the collagen highly expressed at the maternal fetal interface is primarily produced by decidual stromal cells (DSCs) and trophoblasts 11,12. But the role of collagen in fetus implantation and pregnancy is still not clear enough. In the present review, the expression and the possible function of collagen under conditions of physiological and pathological pregnancy are discussed.

## The collagen superfamily

Collagen is one of the important components of ECM in mammals. To date, the collagen superfamily comprises 29 different members numbered with Roman numerals (I-XXIX) 6. Obviously, collagen is widely distributed and expressed in different tissues and organs of the body. For each collagen type, it is consisted of three polypeptide chains, called α chains. These three α chains can be either identical to form homotrimers or different to form heterotrimers. The three α chains of collagen are three left-handed polyproline II helices twisted in a right-handed super helix structure 7. Each distinct α chain is encoded by a different gene. For example, the α chain of collage type IV is encoded by *COL4A1, COL4A2, COL4A3, COL4A4, COL4A5* and *COL4A6* 13-15. In addition, it is possible for a single collagen type to have multiple chain compositions, such as collagen type IV can be composed of three distinct heterotrimeric molecules: [(a1(IV))_2_a2(IV)], [a3(IV)a4(IV)a5(IV)] and [(a5(IV))_2_a6(IV)] 16. The collagen molecule is comprised of a triple helical region and two nonhelical regions at both ends of helix 7. According to the different structure and function, collagen can be divided into different categories, including fibril-forming collagen (e.g., collagen type I and III); basement membrane collagen (e.g., collagen type IV); beaded filament-forming collagen (e.g., collagen type VI); anchoring fibril-forming collagen (e.g., collagen type VI); fiber-associated collagens (e.g., collagen type IX); hexagonal network-forming collagens (e.g., collagen type X); transmembrane collagens (e.g., collagen type XIII); and multiplexins (e.g., collagen type XVIII) 17.

Like other proteins, collagen is synthesized on rough endoplasmic reticulum and transported into Golgi apparatus for processing and then secreted to extracellular. The degradation of collagen is mainly associated with the expression and activity of matrix metalloproteinase (MMP), which are able to degrade different components of the ECM 17. Therefore, MMP plays a crucial role in physiological and pathological processes, such as tissue repair and remodeling, tumor development and metastasis 18,19. Various collagen types are degraded by different MMP. For example, collagen type I, II and III are degraded by MMP-1, MMP-8, MMP-13 and MMP-14. Whereas collagen type IV is preferentially cleaved by MMP-2 and MMP-9 7.

The basic function of collagen is to maintain the structural integrity of tissues and organs. In addition, collagen plays critical roles in organ development, tissue repair, tumor occurrence and metastasis 20-23. Collagen plays a series of roles by binding to different receptors. At present, there are four main types of collagen receptor in vertebrates, including integrin, tyrosine kinases receptor, immunoglobulin-like receptor and mannose receptor 7,24. Furthermore, some of the fragments resulting from the cleavage of collagens are also ligands of integrins 25.

The arrangement of collagen molecules in self-assembled fibers is very important for the biological function of cells attached to them. At the cellular level, the interaction between cells and the surrounding ECM has a critical influence on cell fate and dominates various cell functions. Cells bind to the surrounding ECM through integrin protein, one end of which is bound to the cytoskeleton in cells, the other end has a clear binding site on collagen fibers and other ECM proteins 26. The increased ECM deposition and protein cross-linking may alter tissue rigidity. Fiber assembly of mature collagen molecules involves intermolecular cross-linking between lysine residues on adjacent collagen molecules. Collagen cross-linking is catalyzed by lysine oxidase and happens between hydroxylated or nonhydroxylated lysine residues in collagen 27. The cross-linking of collagen will increase the rigidity and mechanical stability of tissue 28,29. In addition, the composition ratio of collagen also affects the rigidity of tissue. The ratio of collagen type I to type III collagen is essential for the functional integrity of various tissues. The increase in the collagen I/III ratio could lead to the increased tissue rigidity. On the contrary, it will increase the elasticity of the tissue 30. This may be due to the different properties and structures of collagen type I and type III. Collagen type I consists of rigid fibers, while collagen type III is considered immature, fragile and elastic 31. Furthermore, previous studies have also suggested that the rigidity of the collagen modulates cell migration, survival and differentiation 32,33.

## The expression of collagen at the maternal fetal interface

### Collagen expression in the trophoblasts

During the establishment of pregnancy, the embryo implants completely into the uterus surrounded by trophoblasts. The villous trophoblast of the human placenta is the epithelial cover of the fetal chorionic villi floating in maternal blood. As shown in **Table 1**, several studies have reported on the expression of collagen in trophoblasts. Fu et al observed that the expression of total collagens in the villi in the first trimester with Masson staining. And they also observed collagen type IV expression in trophoblasts by RT-PCR and ELISA 12. In addition, trophoblasts are able to secret collagen type IV, and even are the main source of collagen type IV 10-12. And the expressions of collagen type I, III, VI were also detected in villi by immunohistochemistry 10. Besides the expression of collagen protein, recent studies are also focused on the expression of collagen fragment and receptor in the trophoblasts. As shown in **Table 2**, the non-collagenous domain 1 (NC1) from α1-3 (IV) and α5 (IV) chain of collagen type IV expressed in the villi. And some villi were devoid of alpha6 (IV) NC1 immunoreactivity while others stained weakly 10. And the integrin heterodimers α_1_ꞵ_1_ and α_2_ꞵ_1_ and LAIR-2 are found to be expressed in trophoblasts 10,34,35. As mentioned above, collagen has many different receptors. Integrin α10, integrin α11, DDR-1 and DDR-2 are all expressed by all trophoblast types 10. In addition, integrin β1 (also known as CD29), a member of integrin family, mediates cell-ECM communication and is also a receptor of collagen 36. Recent researches also detected the expression of integrin β1 in trophoblasts 37,38.

### Collagen expression in the decidua

Decidual tissue is a special tissue stimulated by decidualization inducer to proliferate and differentiate in endometrial stroma, which is very important for the establishment and maintenance of pregnancy 39. Previous studies showed that collagen type I, III, and V are the main components of ECM in the mouse decidua 40, 41. The expression of total collagens was observed in the human decidua in the first trimester with Masson staining 12. The expression of collagen type I and III, V, VI were also detected in the decidua 10,42,43. Fu et al observed that collagen type IV expressed in DSCs 12. Oefner et al observed that the expression of collagen types IV is upregulated at the maternal fetal interface in the first trimester compared to the proliferative and secretory endometrium, especially highly expressed in the decidua 10. And DSCs could also secret collagen type IV 11,12. There is also a selective presence of collagen type IV NC1 domains in the decidua. The NC1 domains from α1-3 (IV), α5 (IV) and α6 (IV) chain of collagen type IV express in the decidua, but α4 (IV) NC1 domain is absent with the placental bed 10. Pollheimer et al showed that both the Collagen type XVIII mRNA and protein express in the first and third trimester decidua, but not in primary trophoblasts 44. In addition, endostatin is derived from the C-terminal, common NC1 of collagen type XVIII, was also expressed in DSCs at the maternal fetal interface, but absent in all trophoblast subtypes. And their study suggests that DSCs produce endostatin 44. Arresten and canstatin are also expressed in the decidua 45. Furthermore, integrin β1 as a receptor of collagen is observed to express on the DSCs 38,46.

### Collagen expression on decidual immune cells

During the normal pregnancy, the numbers of uterine immune cells increase dramatically, and decidual immune cells consist of NK cells (~70%), macrophages (~15%), T cells (~15%), and a very small number of other types of immune cells 47. The composition of DICs is different from that of peripheral blood. To date, few studies have reported the expression level of collagen on decidual immune cells (DICs) at the maternal fetal interface. A study has reported that decidual NK (dNK) cells can secreted a little of collagen type IV 12. And it is not clear whether collagen is expressed on the other DICs. But it is worth noting the expression of the collagen receptor on the decidual immune cells.

Leukocyte-associated immunoglobulin like receptors (LAIR) -1 acts as an inhibitory receptor of many types of immune cell, which is also a receptor of collagen. LAIR-1 expresses on the dNK, T-cells and macrophages at the maternal fetal interface 11,12,35. And the expression of LAIR-1 on dNK cell is higher than that on peripheral NK (pNK) cell 12. Our previous study has reported that integrin β1 is highly expressed on dNK cell. And the expression of integrin β1 is higher on dNK cell than pNK cell 48. In addition, the expression of integrin β1 is also detected on decidual T cell 49. Integrin β1 is expressed on the macrophage, but it is not clear whether it is expressed on decidual macrophage 50.

These studies mentioned above may provide us with a new idea, that the collagen plays a series of roles at the maternal fetal interface through interacting with its receptor on DICs, and then contributes to successful pregnancy.

### Collagen expression in fetal membranes

The fetal membranes are not part of the maternal fetal interface, but collagen metabolism in that plays a key role in pregnancy, so we also discussed the expression of collagen in the fetal membranes. Human fetal membranes are composed of the amnion and chorion. The main source of tensile strength of the fetal membranes derives from the collagen contents in the amniotic membrane 51. The compact layer of the amnion is mainly composed of collagen type I and type III together with small amounts of collagen type IV, V and VI 52,53, whereas collagen type Ⅳ and V are the major component of basement membrane of amnion 54-56. The expression of collagen type IV is also observed in amniotic fluid 57. Few studies have reported the expression level of collagen in amniotic fluid, although some studies have shown that MMP-9 is expressed in it 58. Due to the source, quantity and composition of amniotic fluid vary with gestational age and the expression of collagen in amniotic fluid has not been systematically described now.

## Regulatory mechanism of collagen expression at the maternal fetal interface

### Pregnancy-associated hormones

During the pregnancy, placental syncytiotrophoblast and corpus luteum could synthesize a variety of hormones, including estrogen, progesterone, and human chorionic gonadotropin (hCG), which play an important role in maintaining normal pregnancy. Although the specific role of pregnancy associated hormones in regulating collagen expression at the maternal fetal interface is not clear, previous studies have reported that estrogen plays a key role in regulating collagen expression in other organs and tissues. Zhou et al proposed that 17β-estradiol and progesterone could inhibit the expression and activity of MMP in IL-1β-stimulated corneal fibroblasts and thus suppressed the collagen degradation in these cells 59. Both collagen type I and III expression were increased after treatment with estrogen in vaginal 60. At present, the research on the regulation of collagen expression by pregnancy-associated hormones in pregnancy mainly focuses on the cervix. Progesterone is able to promote the expression of* COL1A1, COL3A1* and *COL5A1*, whereas estrogen could promote *COL3A1* and *COL5A1* expression in cervix 61. In the mouse uterus, the collagen I, III, VI fibrils became thicker and longer after treatment with 17β-estradiol or progesterone. And the expression of collagen type IV in luminal epithelium basement was reduced after 17β-estradiol treatment 62. It worth noting that Collagen type IV expression in the luminal epithelium (LE) basement membrane is much lower than in the vasculature, whereas, in oil-treated ovariectomized uterus, the collagen type IV staining in the LE basement membrane is equally intense as that in the vasculature 62. As mentioned above, the expression of collagen at the maternal fetal interface is highly in pregnancy. Thus, we speculate that collagen expression at the maternal fetal interface is regulated by pregnancy associated hormones. However, it is unclear whether pregnancy associated hormones modulate collagen expression by regulating its synthesis or degradation, and its specific mechanism needs further research.

### Hypoxia

Hypoxia plays a critical role in immunity and inflammation under both physiological and pathological conditions 63,64. During the first trimester of a normal pregnancy, the maternal fetal interface is exposed to a physiological hypoxia microenvironment, due to the extravillous trophoblasts (EVT) invade the spiral arteries, which form an embolism that blocks the spiral arteries and prevents maternal blood into the feto-placental interface 65,66. Hypoxia triggers a profound change in gene transcription, and hypoxia-inducible factor-1α (HIF-1α) is a transcription factor that widely exists in mammals and human under the condition of hypoxia which is a key factor in response to hypoxia stress 67. There is no direct report on the relationship between hypoxia microenvironment and collagen expression at the maternal fetal interface. However, it has been reported that collagen is regulated by hypoxia in other tissues or cells. A study has reported that maternal hypoxia significantly increased collagen type I expression in the neonatal heart by western blotting 68. The expression level of collagen type XV is greatly increased in hypoxia-preconditioned human mesenchymal stromal cells (hMSCs). And HIF-1α activity might play a role in collagen type XV transcriptional activation in hMSCs 69. The expression of collagen type I and HIF-1α both increase in hepatic stellate cells (HSCs) under the condition of hypoxia 70,71. COL1A1 and COL3A1 protein expressions are increased in hypoxia HSCs compared to normoxia group. And the expression of HIF-1α is also increased. Importantly, the deposition and secretion of COL1A1 and COL3A1 are decreased by silencing HIF-1α expression 72. To sum up, under the condition of hypoxia, there may be an increase of HIF-1α expression, thus promoting the expression of collagen. In addition, some diseases occur during pregnancy may lead to the changes of hemodynamic parameters of placental bed artery. And abnormal blood flow in the intervillus is able to affect the diffusing function of the placental barrier, and then create a state of hypoxia. Ortega et al found that the increased presence of collagen type III in the placenta of women with venous insufficiency compared to normal group 73. This result suggests that the expression of collage0n0… increase here may be a mechanism triggered by a possible hypoxia state caused by altered blood flow in the placenta in patients with venous insufficiency during pregnancy. To date, there is insufficient experimental evidence to prove that collagen expression at the maternal fetal interface is directly regulated by hypoxia, but it is known that the maternal-fetal itself is a hypoxic microenvironment, and the expression of some collagen is increased during pregnancy. Therefore, we speculate that the hypoxia microenvironment at the maternal fetal interface may be able to promote the expression of collagen.

## The role of collagen in normal pregnancy

### Trophoblast invasion

Trophoblasts exert a critical role in normal pregnancy. Their proliferative, invasion, and apoptotic properties are modulated by a series of factors, including cytokines, chemokines, ECM and so on 74,75. As mentioned above, the expression of collagen and its fragment could be observed at the maternal fetal interface. However, the effect of collagen protein and collagen fragments seems to be the opposite. Traditionally, the collagen has been thought of primarily as a three-dimensional scaffold that binds cells and tissues together 76. However, collagen also is able to regulate the biological behavior of cells. Collagen protein plays a critical role in enhancing the adhesion, proliferation and invasion of cell 77-79. Collagen has an important role in trophoblast adhesion at the maternal fetal interface. It was observed that trophoblast adhesiveness was highest in the presence to collagen type I and collagen type IV compared with other ECM 80. Collagen type I enhances carcinoma cell migration* in vitro* 81. And collagen type XVII could promote less aggressive squamous cell carcinoma cells invasion 82. However, some of the NC1 domain of collagen seem to have the ability to inhibit angiogenesis, cell proliferation and invasion.^30^ Recent studies have reported that endostatin which is the NC1 domain of collagen type XVIII could restrict the development of tumor by inhibit the proliferation, invasion of cell 83,84. The α6 (IV) and α4 (IV) domain of collagen type IV exert an anti-tumor activity by decreasing proliferative and invasive properties of tumor cell 85. At present, there are not enough studies reported on the role of collagen and its fragments in pregnancy. It is worth noting that collagen and its fragments are both expressed at the maternal fetal interface, although their expression levels are not consistent. Trophoblast could cleave endostatin and endostatin-like fragments from NC1 domain of collagen type XVIII by producing proteases 86. Recombinant endostatin reduces trophoblast invasion *in vitro* 86. A previous study has suggested endostatin upregulates MMP-2 expression in trophoblasts in order to modulate trophoblast invasiveness 44. Pollheimer et al proposed that invasive trophoblasts may produce the inhibitory protein as encountering stromal cells of the decidua basalis 86. In addition, EVT expresses integrin αvꞵ3, which can bind to α2 (IV) and α3 (IV) NC1 domains 87,88. These results suggest that NC1 domain of collagen type IV may have a direct interaction with trophoblast. So we speculate that the α (IV) NC1 domain of collagen type IV which is selectively expressed at the maternal fetal interface may similarly influence MMP expression by EVT, and then regulate the invasion of trophoblast. Furthermore, integrin ꞵ1 participates in the trophoblast invasion, but whether collagen is involved in this process has not been confirmed yet 37,38.

The degradation and remodeling of collagen in the uterus in mice during the peri-implantation should be given attention. In the preimplantation period, collagen type I, III, IV and VI were observed throughout the uterine endometrium and decidua. During the peri-implantation, the expression of collagen type I, III, VI disappear from the decidua upon embryo. Whereas, collagen type IV accumulates in the decidua, and also expressed highly in embryonic basement membranes 62.

**Figure 1:**
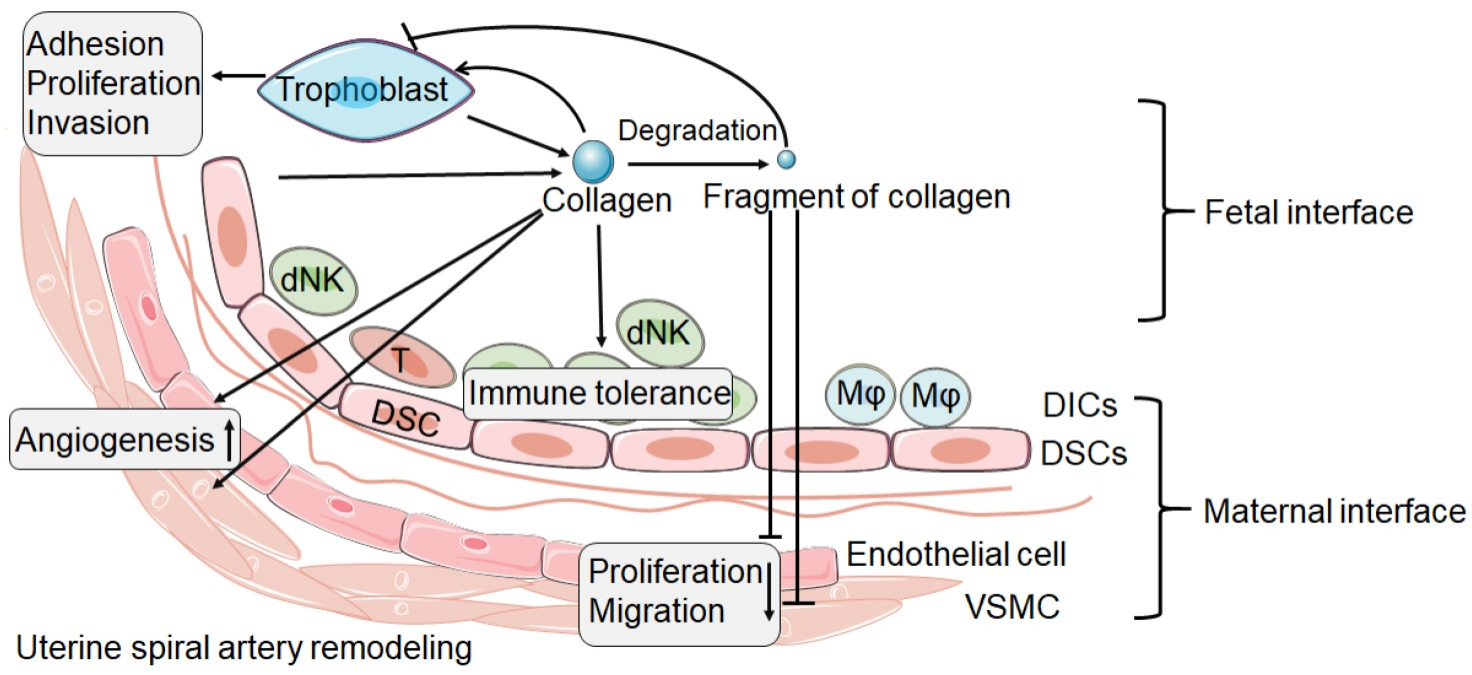
** The possible role of collagen and its fragment at the maternal fetal interface.** The collagen at the maternal fetal interface is mainly produced by trophoblast and decidual stromal cell (DSC). Collagen might play an important role in the trophoblast adhesion, proliferation and invasion. But the process of trophoblast invasion is accompanied by collagen degradation and remodeling, and then produces a number of collagen fragments. The fragment of collagen might be able to inhibit the trophoblast invasion. In addition, collagen and its fragment seem to have the opposite effect in spiral artery remodeling. Collagen may promote the spiral artery remodeling. However, the fragment of collagen seems to be able to inhibit the proliferation and migration of vascular smooth muscle cell (VSMC) and endothelial cell. In addition, collagen promotes immune tolerance of decidual immune cells (DICs). dNK: decidual natural killer cell; T: T cell; Mφ: macrophage.

During the establishment of pregnancy, placentation involves in the invasion of decidua basalis and the inner third of the myometrium by EVT. It is known that this process requires trophoblast pass through ECM and remodeling of ECM. Collagen as an important component of ECM, which is highly expressed at the maternal-fetal interface, must undergo remodeling during trophoblast invasion 89. Based on the current research, we speculate that, as shown in** Figure 1**, integrin expressed by trophoblast combines with collagen expressed at the maternal fetal interface to promote trophoblast adhesion. The trophoblast then invades into decidual tissue, which is accompanied by collagen degradation and remodeling. The degradation of collagen produces different fragments, which in turn inhibit the invasion of trophoblasts, thus achieving a delicate balance.

### Immune tolerance

Pregnancy is an immune paradox in which the semi-allogenic fetus must not be rejected by the maternal immune system 90. The mechanisms that underlie in forming immune tolerance microenvironment are of critical importance, some factors expressed at the maternal fetal interface contribute to the immune tolerance, such as collagen. Previous study has reported that collagen has potent immunomodulatory properties in autoimmune and tumor diseases 91,92. The forming and maintenance of immune tolerance at the maternal fetal interface are accompanied by special dNK cells function, T helper cell 2 (Th2) bias and macrophage polarization.

As mentioned above, the numbers of DICs increase dramatically during decidualization, and the composition of DICs is different from the leukocytes in the peripheral blood. It is believed that changes in the DCIs are partly due to the recruitment of peripheral immune cells, and partly due to the resident population of endometrium 93,94. Previous studies have reported that chemokine could regulate the recruitment of DICs. Besides the recruitment of immune cells, the residence of DICs is also very important for maintaining immune tolerance during normal pregnancy. It is reported that integrin ꞵ1 is important for the adhesive ability of dNK cells to DSCs 48. Due to the DSC is observed to highly express collagen, and collagen could regulate the adhesion by integrin ꞵ1 in other cells, collagen is supposed to be involved in the adhesion between dNK and DSC 95. Thus, we proposed that collagen expressed at the maternal fetal interface is important for NK cell residence by integrating with integrin expressed on dNK cells.

**Figure 2:**
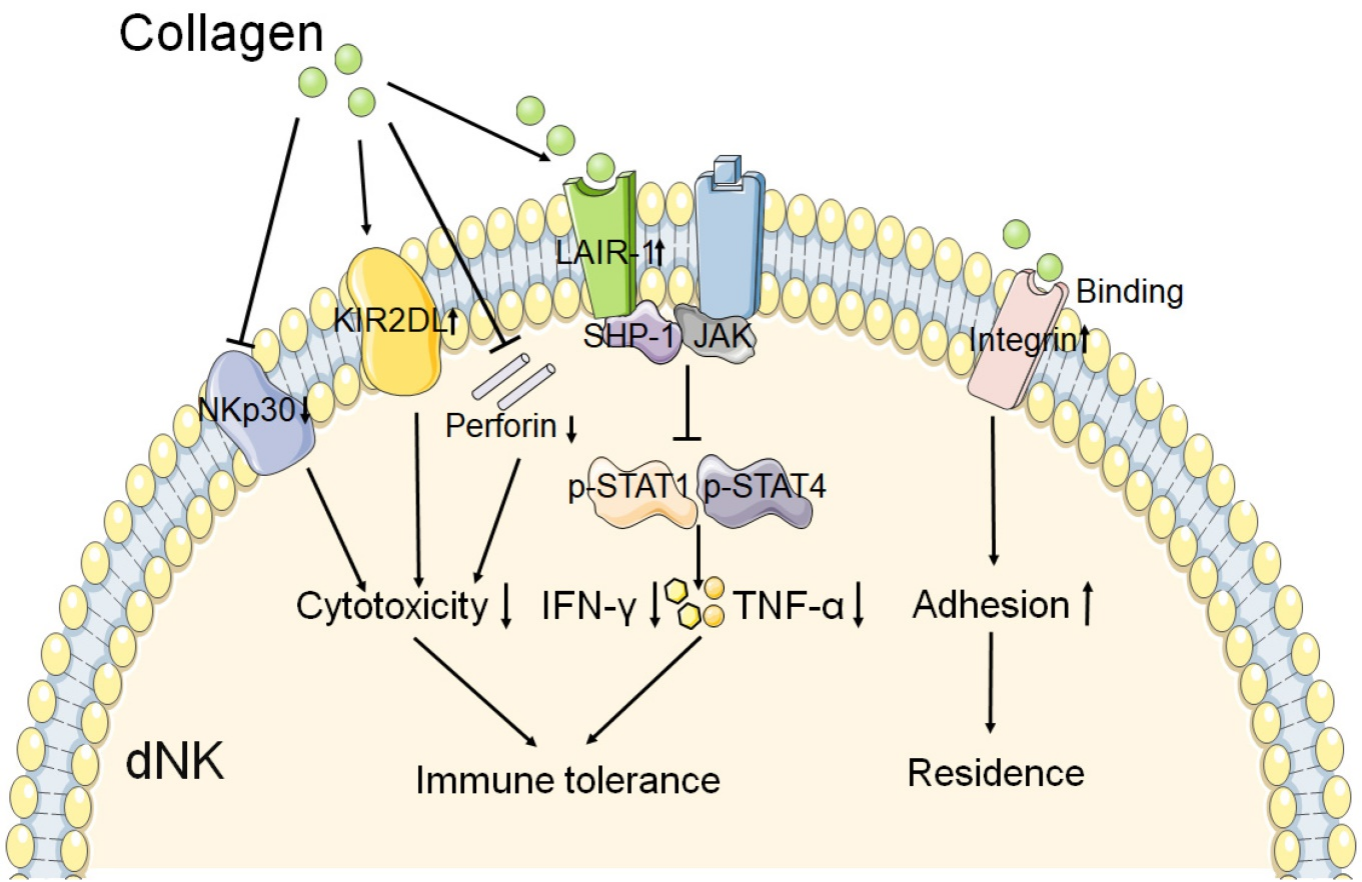
** The role of collagen in the function of dNK cell.** The interaction of collagen with integrin β1 may promote the adhesion of dNK cell, and then contribute to the residence of NK cell in decidua. And collagen binds to the LAIR-1 which is expressed on dNK cell, triggers the SHP-1 binding to JAK1 and JAK2 in dNK cell. Resulting in the phosphorylation of STAT1 and STAT4 reduction, and then the production of IFN-γ and TNF-α is decreased. In addition, collagen could reduce the cytotoxicity receptor NKp30 expression, and increase the inhibitory receptor KIR2DL1 expression on dNK cell. The production of perforin is also decreased by collagen. These results mentioned above leads to decreased cytotoxicity and activity of dNK cell. Therefore, collagen plays a key role in the function of dNK cell, and then contributes to the immune tolerance at the maternal fetal interface.

A study has reported that collagen could decrease the expression of NKp30 on dNKs, one of the natural cytotoxicity receptors, and perforin 12. This result suggests that collagen might contribute to the decreased cytotoxicity of dNK at the maternal fetal interface. The expression of inhibitory receptor KIR2DL1 on dNKs was increased after treatment with increasing concentrations of collagen 12. Fu and his team observed that trophoblasts and DSCs could regulate the expression of IFN-γ and TNF-α in dNK cells through collagen. And the results of western blot show that collagen could upregulate the expression of LAIR-1, decreased the expression of both p-STAT1 and p-STAT4 in dNK cells 11. So they proposed that collagen which produced by DSCs and trophoblasts induced LAIR-1 expression. And then interaction between collagen and LAIR-1 triggers the binding of SHP-1 to JAK1 and JAK2 in dNK cells, and this leads to downregulation of the phosphorylation of STAT1 and STAT4 11. As a result, the production of IFN-γ and TNF-α is inhibited, which contributes to the immune tolerance at the maternal fetal interface. As shown in **Figure 2**, these results mentioned above suggest that collagen play a key role in immune tolerance by regulation the function of dNK cells during normal pregnancy.

There is no direct report on the role of collagen in regulating T cell differentiation and macrophage polarization at the maternal fetal interface. However, as mentioned above, integrin ꞵ1 and inhibitory receptor LAIR-1 express on the decidual T-cells and macrophages at the maternal fetal interface 11,12,35,49. And recent research suggested that collagen type II is able to induce peripheral tolerance in *BALB/c* mice by generating CD8^+^ T regulatory (Treg) cells 96. Polymerized-type I collagen has been shown to exert downregulation of autoimmune inflammation by regulating both Treg/Th17 differentiation and Th1/Th2 balance 97. During the development of collagen-induced arthritis, the collagen-specific CD4^+^ T cell response shifts *in vivo* from a dominant Th0/Th1 response to a clear Th2 phenotype 98. Therefore, collagen may play a key role in T cell differentiation at the maternal fetal interface.

In addition, previous studies have suggested that decidual macrophages, which play a critical role in the endometrium during normal pregnancy, are more closely associated with the M2 phenotype 99,100. Chen et al observed that lack of collagen type VI in Col6al^-/-^ mice increased polarization toward the M1 phenotype, and reduced polarization toward the M2 phenotype 101. In addition, Lv et al observed that collagen type VI within the nerve conduit could induce polarized macrophage toward the M2 phenotype, and then promote nerve regeneration and functional recovery 102. These results suggest that collagen is able to induce the polarization of M2 in other tissues, which provides the possibility for the future study of collagen promoting the differentiation of macrophages into M2 phenotype at the maternal fetal interface.

In conclusion, the high expression of collagen at the maternal fetal interface may induce the immune tolerance microenvironment by regulating the differentiation and function of immune cells in decidua, which is conducive to successful implantation and pregnancy.

### Angiogenesis

Angiogenesis is a key process during a successful pregnancy, which is involved in decidualization, implantation, and embryo growth 103. During the process of angiogenesis, ECM remodeling is particularly important for the development of new blood vessels. Collagen, as the primary constituent of the ECM, requires active degradation and remodeling as endothelial cells proliferation, migration and differentiation, in order to form new vascular networks. The current research is mainly based on collagen as a three-dimensional scaffold to support angiogenesis 104-106. Previous study has suggested that collagen type VI began to clear in the decidua during implantation 62. Interestingly, the degradation of collagen type VI in the decidual does not extend to the vessel walls 62,107. It probably suggests that collagen which envelops vessels could integrate them into the three-dimensional tissue architecture in order to vascular remodeling. In addition, the fragment of collagen seems to have the ability to inhibit angiogenesis. Three separate α-chain trimers exist for type IV collagen: [(a1(IV))_2_a2(IV)], [a3(IV)a4(IV) a5(IV)] and [(a5(IV))_2_a6(IV)]. Since collagen type IV consists of six different α chains, the degradation of collagen type IV can potentially give rise to a vast array of fragments. Some of the NC1 fragments of collagen type IV have anti-angiogenic effects that decrease endothelial cell proliferation, migration and tube formation, as well as increase endothelial cell apoptosis, including arresten (COL4A1), canstatin (COL4A2) and tumstatin (COL4A3) 30,108-110. Previous study reported that overexpression of arresten in vascular smooth muscle cells (VSMC) could reduce VSMC proliferation 111. VSMC also play a critical role in spiral artery remodeling process in pregnancy. Therefore, the NC1 fragment of collagen may be involved in regulating the physiological remodeling of maternal spiral arteries. In addition, as mentioned above, endostatin expression was observed at the maternal fetal interface. The role of endostatin in uterine spiral artery remodeling has not been reported. But endostatin can reduce endothelial cell migration, proliferation, and then inhibit vascular angiogenesis in other tissues 112-115. Based on the current research, it can be concluded that the collagen and its fragments expressed at the maternal fetal interface may be able to regulate the remodeling of spiral arteries, and collagen metabolism may also be closely related to vascular remodeling.

In summary, we speculate that the collagen expressed at the maternal fetal interface may play a role in promoting trophoblast invasion and angiogenesis. MMP selectively degrade collagen and produce fragments which may exert to inhibit cell adhesion, proliferation and invasion, as well as have anti angiogenic effect. There is a delicate balance between the expression of collagen protein and collagen fragments, which contributes to successful pregnancy.

### Cervical remodeling

Cervical remodeling is accompanied by extensive collagen metabolism, which is essential for successful pregnancy. During normal pregnancy, the cervix keeps closed and firm in order to prevent passage of an immature infant through the birth canal. On the contrary, at term, the cervix must become soft and open sufficiently to allow delivery of the full-term fetus. The cervix stroma is mainly composed of fibroblasts, followed by smooth muscle cells 116. These cells can secret a series of ECM, including collagen fiber, elastin and so on. During pregnancy, cervical remodeling is the process of a series of physiological changes in the cervix, which can be divided into four stages: softening, ripening, dilation and postpartum repair, all of which are accompanied by ECM remodeling 117. Collagen type I and III are the main components responsible for mechanical strength in the cervix 27,116. Due to the limitations of human *in vivo* experiments, research of cervical remodeling in pregnancy is mainly from rodent experiments. The expression of collagen type I in the cervix during the first half of pregnancy was similar to the nonpregnant mice, but significantly increased in the latter days of gestation. And then levels declined to nonpregnant group by 24 hours postpartum 27. Previous studies have demonstrated that the cervical softening is characterized by an increase in the solubility of collagen and no change in the total collagen content 117. That is to say, the decrease of collagen cross-linking forming enzyme leads to the decrease of cross-links between collagen molecules, and then reduces the mechanical strength of cervical tissue 27,118. Unlike cervical softening, the ripening process is very rapid, during which the compliance of the cervix increases and the cervix is easy to dilate. At this stage, the diameter of cervical collagen fibrils gradually increases, and the distance between fibers also increases 118. In addition, the characteristics of this stage also include the transition from long and thin collagen fibers to thick and curved fibers in the cervix 118. These changes enable the cervix to maximize its compliance and thus facilitate cervical dilatation. The causes of spontaneous preterm birth are very complex. One of the reasons is that cervical softening, shortening and dilation caused by early cervical remodeling can lead to spontaneous preterm birth. Progesterone treatment could reduce the risk of premature birth 119,120. Prepartum cervical ripening is closely related to collagen remodeling in cervix. Progesterone withdrawal could reduce the abundance of collagen fibers in the cervix, and promote the cervical remodeling in nonpregnant mice 121. Steven et al reported that the density and structure of collagen in cervix were enhanced by treatment with progesterone 122. It has been reported that the progesterone receptor proteins in the cervix of women prior to parturition decreased significantly 52. These results suggest that the concentration of progesterone is associated with collagen remodeling in cervix. Progesterone treatment may reduce the occurrence of premature birth through delaying cervical remodeling.

## The role of collagen in pathological pregnancy

### Recurrent miscarriage

Recurrent miscarriage (RM), also known as spontaneous abortion, refers to the experience of at least two or three spontaneous miscarriages before the 24th gestational week. Chromosome and endocrine abnormalities, anatomical defects, acquired infection, and immunological factors are understood to be the major cause of RM 123,124. The cause of RM remains unexplained in almost half of patients. A series of studies have shown that the abnormal expression of collagen and its fragments at the maternal fetal interface may be associated with unexplained recurrent miscarriage. The staining of total collagens in the villi and decidua is weaker in miscarriage compared with normal pregnancy by using Masson staining 12. Decreased expression of collagen is observed in decidual tissue of patients who undergo RM, including collage type IV and collagen type V 11,12,42,43. Although the α chains of collagen type I and III were almost similar in the decidua obtained from normal pregnancy and RM, those of α1 (V) and α2 (V) were markedly decreased in the decidua from RM 42. In addition, the mRNA and protein secretion of collagen type IV in trophoblasts and DSCs from miscarriage were lower than those of normal pregnancy 12. It is not clear whether the abnormal expression of collagen is the cause or the result of miscarriage. But as mentioned above, collagen has an important role in inducing immune tolerance at the maternal fetal interface. And there is also abnormal expression of collagen at the maternal fetal interface. In addition, the expression of LAIR-1 on dNK cells from miscarriage was decreased compared with that of the normal pregnancy 12. However, the expression level of integrin ꞵ1 is higher on dNK cells from RM than those from women with normal pregnancy 48. This result suggests that the dNK cells form RM have stronger adhesive capacity. But the relationship between abnormal expression of integrin ꞵ1 and the development of RM has not been revealed. Based on the studies mentioned above, we speculate that the abnormal expression of collagen at the maternal fetal interface may not fully form the immune tolerance microenvironment, leading to miscarriage. Future research can focus on the role of collagen at the maternal fetal interface immune microenvironment, so as to provide a new target for RM treatment.

### Diabetes mellitus with pregnancy

There are two conditions of diabetes occur during pregnancy, one is diabetes mellitus with pregnancy, and another is gestational diabetes. Compared with women who do not have diabetes mellitus, those with pre-existing diabetes mellitus have an increased risk of pregnancy complications. Previous researches reported impaired decidualization in diabetic mouse 125,126. Dixon et al observed in the diabetic nephropathy, the expression levels of collagen types IV and V increase compared to normal people, whereas collagen types I and III appear at advanced stages of the disease 127. Favaro and his team established a mouse model of long-term type 1 diabetes specifically delineated to reproductive investigations. The model mouse has impaired decidua and less implantations compared to the control group. And they found that the deposition collagen increased in the decidua in the model group compared to the control group. In addition, they analyzed the contribution of specific collagen types through immunohistochemistry. Diabetes increased the proportion of collagen types I and V, whereas decreased collagen type III deposition. And in the expression level of mRNA, only *COL1A1* mRNA levels significantly increased in the diabetic group decidua 126. Above results indicate that collagen composition at the maternal fetal interface is mainly regulated by post transcriptional mechanisms in the diabetic pregnancy. The defect of decidualization is related to the abnormal deposition of type III collagen 128. Therefore, depended on the researches mentioned above, impaired decidualization by diabetes might be associated with abnormal expression of collagen at the maternal fetal interface during early embryonic development.

### Preeclampsia

Preeclampsia is a serious complication of pregnancy manifested as maternal hypertension in pregnancy (HTN-Preg) and fetal growth restriction, and also a major contributor to maternal and perinatal morbidity and mortality worldwide 129. Inadequate placentation and placental ischemia could be initiating events of the occurrence of preeclampsia. Placental ischemia might be an important factor leads to abnormal vascular remodeling at the maternal fetal interface. Placental ischemia, possibly through the production of TNF-α, contributes to the increasing levels of MMP-1 and MMP-7, which, in turn, alter collagen deposition and cause inadequate tissue remodeling in HTN-Preg 130. In turn, the over deposition of collagen may lead to inadequate vascular remodeling at the maternal fetal interface, resulting in pregnancy-induced hypertension and even preeclampsia. Lin et al observed that the collagen type IV immunoreactive bands were enhanced in uterus and uterine artery of reduced uterine perfusion pressure (RUPP) compared to pregnant rats 9. Compared with normal pregnant rats, the MMP-2 and MMP-9 levels in the aorta, uterus and placenta of RUPP rats decreased, suggesting that low MMP levels may lead to excessive collagen deposition, affect smooth muscle growth, and decrease the remodeling of spiral arteries 131. Therefore, the abnormal expression of MMP may lead to the over deposition of collagen, which may affect the remodeling of uterine spiral artery, and it may be an important factor in the pathogenesis of preeclampsia.

In addition, there is abnormal expression of collagen fragments in preeclampsia. As mentioned above, the polypeptide chains of Collagen type IV are encoded by six genes, including *COL4A1, COL4A2, COL4A3, COL4A4, COL4A5* and *COL4A6*. A study identified that* COL4A1* and *COL4A2* as maternal preeclampsia susceptibility genes, which code for collagen type IV α chain 1 and α chain 2 132. And the mRNA expressions of these genes are significantly increased in preeclampsia decidua 133. As mentioned above, arresten is considered to be an anti-angiogenic factor. Additionally, Yong et al. demonstrated that the expression of arresten fragments were significantly increased in plasma during the second and third trimester in the preeclampsia compared with normotensive. The increase in arresten during the third trimester correlated with the requirement for magnesium sulphate treatment. In addition, decidual levels of arresten monomer were also increased in the third trimester 133. The role of abnormally expression of arresten in the pathogenesis of preeclampsia is not clear. But previous study reported that arresten on primary vascular smooth muscle cells (VSMC) by transfecting the cells with the arresten gene, could reduce VSMC proliferation 111. And VSMC plays a critical role in spiral artery remodeling process in pregnancy. Therefore, arresten, which is the NC1 fragment of collagen type IV, may be involved in regulating the function of VSMC, and then mediating the occurrence of preeclampsia. Based on the above study, we speculate that the over deposition of collagen and some fragments of collagen may be related to the insufficient remodeling of uterine spiral artery at the maternal fetal interface.

Human placenta is an important organ for fetal growth and development, which consists of the basal decidua of the maternal components and chorionic villi of the fetus. Previous studies suggest that fibrosis in the villous stroma of preeclampsia placentas is associated with ischemia and hypoxia 134,135. During early placental development, the invasion of extravillous trophoblast into placental bed is insufficient, which leads to the dysfunction of uterine spiral artery remodeling. In turn, this causes the decrease of perfusion of intervillous space. In this ischemic and anoxic environment, villi undergo a series of changes, such as collagen deposition 134. Fibrosis of villous stroma is one of the most prominent features of preeclampsia, which refers to the excessive deposition of ECM in the connective tissue 134. As mentioned above, the abnormal expression of collagen in placenta tissue could be observed in the patients of preeclampsia. And the level of COL1A1 is higher in the fibroblasts from preeclampsia than those from normal placentas 136. Placental fibroblasts could increase the production of collagen type I and IV in hypoxic conditions in *vitro* 64,136. A previous study suggests that due to ischemia and hypoxia, transforming growth factor β1 (TGFβ1) signal is activated in fibroblasts of preeclampsia placenta, which may lead to the over production of collagen, and then contributes to placenta fibrosis 136.

In conclusion, in the placenta of preeclampsia patients, the over-activation of fibroblasts and the decrease of MMP expression lead to the abnormal deposition of collagen at the maternal fetal interface, and promote placental fibrosis. In turn, excessive deposition of collagen will lead to the insufficient remodeling of uterine spiral artery, and aggravate the progress of preeclampsia.

### Premature rupture of membranes

The rupture of fetal membranes is a crucial event in parturition, accompanied with extensive degradation of collagen. Premature rupture of membranes (PROM) refers to the spontaneous rupture of fetal membranes before delivery. Full-term PROM refers to the premature rupture of membranes occurring in woman after 37 weeks of pregnancy. Preterm premature rupture of membranes (pPROM) accounts for about one third of preterm birth 116. The physical integrity of the fetal membranes is required to be maintained until term delivery in normal pregnancy. The animal model has demonstrated that the amnion undergoes collagen remodeling as gestation progress 137. Previous studies indicate that with advancing gestational age, the abundance of collagen in the fetal membranes decreases 138,139. COL1A1 and COL1A2, the two subunits of collagen type I, which is one of the major collagen types that determine the tensile strength of the amnion, are significantly decreased in the vaginal delivery with amnion tissues of spontaneous rupture of membranes as compared with the amnion tissues from pregnancies after elective cesarean section without rupture of membranes and labor 140. Zuo et al reported that the expression of collagen type IV in full-term PROM patients in fetal membranes was significantly less than the control group. And they also observed that serum collagen type IV in full-term PROM patients was lower, whereas serum and amniotic fluid MMP-9 were higher than control group 58. It is known that collagen type IV is one of the main substrates of MMP-2 and MMP-9. Thus, the authors suggested that the serum increased serum MMP-9 induced the degradation of collagen type IV, and thus led to the PROM 58. And detection of MMP and collagen expression in serum may be able to predict the risk of PROM in pregnant women in advance. Theoretically, the increase of MMP-9 in amniotic fluid of pregnant women with PROM will result in the decrease of collagen type IV in amniotic fluid. However, unexpectedly, the collagen type IV of amniotic fluid in full-term PROM patients was significantly higher than that of the control group 58. This result suggests that except for MMPs, collagen type IV may be regulated by other factors. Previous study has demonstrated that the activity of MMP-2 and MMP-9 in amnion increased with gestational age 141. The expression and activity of MMP-2 and MMP-9 in the fetal membranes is increased markedly in labor compared with nonlabor, whether in term or preterm 139,141. In addition, the abundance and activity of MMP-7 in the amniotic fluid and amnion tissue increased with the increase of gestational age and further increased in preterm labor 142,143. Collagen type IV is the major component comprising the basement membrane in the amnion, and also is the most important substrate of MMP-7. Maymon et al suggests that the abundance of MMP-7 in the amniotic fluid is increased with the increase of gestational age and further increased in preterm labor 142. Wang et al observed that the expression of MMP-7 was increased, whereas COL4A5 was decreased significantly in the amnion tissue collected from vaginal deliveries with spontaneous rupture of membranes compared with that collected from elective Caesarean sections without labor 144. This data suggests that collagen degradation by MMP leads to the weakening of the strength of the fetal membrane, and consequently results to the rupture of the fetal membranes. The degradation of collagen can occur either extracellularly through degradation by MMPs or intracellularly via autophagic and proteasomic pathways in the process of membrane rupture. Cortisol regenerating enzyme 11β-hydroxysteroid dehydrogenase 1 (11 β-HSD1) is expressed in almost all cell types of fetal membranes, and its expression increases with the advancing of gestational age 145,146. This result can explain the increase of cortisol abundance in fetal membranes in the last trimester. Cortisol regenerated by 11 β-HSD1 in amniotic fibroblasts is able to reduce the abundance of collagen type III by ubiquitin proteasome pathway 52. And collagen type I could be degraded by cortisol through lysosome-mediated autophagy in amnion fibroblasts 147. These results suggest that cortisol mediated degradation of amniotic collagen may play an important role in fetal membranes rupture.

Additionally, the MMP/TIMP imbalance is also involved in the fetal membranes rupture. Tissue inhibitor of metalloproteinase (TIMPs) is an endogenous secretory protein, which is a specific inhibitor of MMPs. TIMPs can bind with zinc ion in the corresponding catalytic activity center of MMP and block its catalytic activity. TIMPs are thought to regulate degradation of basement membrane and ECM by MMPs during tissue remodeling. Previous reports have observed the presence of TIMP-1, TIMP-2, TIMP-3 and TIMP-4 in fetal membranes collected from women undergoing normal labor as well as nonlaboring women 148. The level of TIMP-1 in amniotic fluid in pPROM group is higher compared with women with preterm and term labor with intact membranes and undergoing Cesarean sections, whereas the expression of TIMP-2 decreased in pPROM 54,140. Similarly, a study also suggests that the concentration of TIMP-2 in amniotic fluid increases with gestational age but decreases in the context of term and preterm labor, rupture of membranes and intrauterine infection 149. These results suggest that the increase of TIMP-2 concentration in amniotic cavity may help to protect amniotic membrane from excessive MMP activity generated during the process of uterine distention. And when in labor, the decrease of TIMP-2 relieved the inhibition of MMP activity, contributing to the rupture of fetal membrane. Previous studies have reported that the expression of MMP and TIMP is regulated by microbial infection in other tissues 150,151. Maymon et al found that amniotic fluid TIMP-2 concentrations were significantly lower in women with intraamniotic infection than in those without infection, whereas infection did not increase amniotic fluid MMP-2 concentrations. But another report showed that the expression of MMP-2 and MMP-9 in the fetal membranes was increased after exposure to Escherichia coli, while the secretion of TIMP-1, TIMP-2, and TIMP-4 remained without significant changes. At the same time, the collagen content was significantly decreased in infected membranes 152. These results indicate that the expression of MMP/TIMP is regulated by pathogenic microorganisms, but different pathogens may affect different types of MMP/TIMP. And the abnormal expression of MMP/TIMP in amniotic fluid may indicate the presence of intraamniotic infection. Infection will affect the balance of MMP/TIMP in amniotic fluid or fetal membrane, which will lead to the degradation of collagen and rupture of fetal membrane, ultimately affect the outcome of pregnancy.

## Conclusions and perspectives

Collagen is one of the most abundant proteins of the ECM. Numerous studies have reported on its role in tumor. However, in recent years, its specific role at the maternal fetal interface has also attracted increasing levels of attention. Some of the collagen protein and its fragments are expressed at the maternal fetal interface, and fulfill a series of roles through interacting with their receptors. On the one hand, collagen might have the ability to regulate the trophoblast proliferation and invasion, and even angiogenesis. On the other hand, collagen exerts important roles in inducing immune tolerance at the maternal fetal interface. In addition, there are abnormal expression levels of collagen in pathological pregnancy. Therefore, at the maternal fetal interface, the normal expression of collagen may be essential for successful pregnancy. However, the mechanism of collagen action at the maternal fetal interface is not clear enough. For one thing, there is a great variety of collagen, and its degradation can also produce numerous fragments. Furthermore, the research on collagen at the maternal fetal interface is limited to some types at present. For another thing, it seems that the effect of collagen and its fragments is not consistent. Furthermore, collagen at the maternal fetal interface is constantly degraded and remolded during the establishment of pregnancy. Thus, regulating the expression and metabolism of collagen may be a potential target for clinical treatment of patients with abnormal pregnancy, although further researches are required to confirm it.

## Figures and Tables

**Table 1:** The expression of collagen and its receptor at the human maternal-fetal interface

	Trophoblast	Decidua	Decidual immune cells		
			NK cell	T cell	Macrophage
Total collagen	+^11^	+[11]	Not mentioned	Not mentioned	Not mentioned
Collagen type I	+^12^	+[12,34]	Not mentioned	Not mentioned	Not mentioned
Collagen type III	+[12]	+[12]	Not mentioned	Not mentioned	Not mentioned
Collagen type IV	+[10-12]	+[10-12]	+[11]	Not mentioned	Not mentioned
Collagen type V	Not mentioned	+[34]	Not mentioned	Not mentioned	Not mentioned
Collagen type VI	+[12]	+[12]	Not mentioned	Not mentioned	Not mentioned
Collagen type XVIII	-[36]	+[36]	Not mentioned	Not mentioned	Not mentioned
LAIR-1	-[27]	Not mentioned	+[10,11,27]	+[27]	+[27]
Integrin	+[12,26,29]	+[30,38]	+[40]	+[41]	Not mentioned

**Table 2:** The fragment of collagen at the human maternal-fetal interface

	Collagen type IV NC1 domains						endostatin	arresten	canstatin
	α1	α2	α3	α4	α5	α6			
Trophoblast	+[12]			-[12]	+[12]	+/-[12]	-[36]	Not mentioned	Not mentioned
Decidua						+[12]	+[36]	+[37]	+[37]
